# Metabolic Dysfunction and Peroxisome Proliferator-Activated Receptors (PPAR) in Multiple Sclerosis

**DOI:** 10.3390/ijms19061639

**Published:** 2018-06-01

**Authors:** Véronique Ferret-Sena, Carlos Capela, Armando Sena

**Affiliations:** 1 Centro de Investigação Interdisciplinar Egas Moniz (CiiEM), Instituto Universitário Egas Moniz, Campus Universitário, Quinta da Granja, Monte de Caparica, 2819-511 Caparica, Portugal; versena@egasmoniz.edu.pt; 2 Centro Hospitalar de Lisboa Central, EPE, Hospital de Santo António dos Capuchos, Departamento de Neurociências, Alameda de Santo António dos Capuchos, 1169-050 Lisboa, Portugal; carlos.capela.trabalho@gmail.com

**Keywords:** multiple sclerosis, metabolism, peroxisome proliferator-activated receptors, immune system, neuroinflammation, neurodegeneration

## Abstract

Multiple sclerosis (MS) is an inflammatory and neurodegenerative disease of the central nervous system (CNS) probably caused, in most cases, by the interaction of genetic and environmental factors. This review first summarizes some clinical, epidemiological and pathological characteristics of MS. Then, the involvement of biochemical pathways is discussed in the development and repair of the CNS lesions and the immune dysfunction in the disease. Finally, the potential roles of peroxisome proliferator-activated receptors (PPAR) in MS are discussed. It is suggested that metabolic mechanisms modulated by PPAR provide a window to integrate the systemic and neurological events underlying the pathogenesis of the disease. In conclusion, the reviewed data highlight molecular avenues of understanding MS that may open new targets for improved therapies and preventive strategies for the disease.

## 1. An Overview of Multiple Sclerosis (MS) 

Multiple sclerosis (MS) afflicts about 2.5 million patients worldwide and is one of the most common causes of permanent disability in young adults. MS is a disease of the central nervous system (CNS) with the most frequent onset in young adulthood and very different clinical courses in individual patients. About 85% of patients develop the relapsing-remitting type of the disease (RRMS), which is more common in women and starts with episodes of clinical symptoms (relapses) with variable recovery. Most of these patients develop secondary progressive MS (SPMS), a stage of progressive disability not associated with relapses. Patients with primary progressive MS (PPMS) have a progressive course from disease onset regardless of eventual relapse episodes. Clinically isolated syndromes (CIS) categorize patients who experienced a first clinical presentation suggestive of MS. Because many of these patients convert to clinically definite MS, some authors include CIS as another element of the MS phenotype spectrum [1].

Histopathological hallmarks of the disease include multifocal lesions of demyelination, inflammation, gliosis and axon loss or damage (the MS plaque). Typically, these lesions are associated with breakdown of the blood-brain barrier (BBB) and are thought to be mainly mediated by type 1 helper T cells (Th1), Th17, and CD8^+^ cells, despite increasing evidence for an early involvement of B cells and the innate immune system (including macrophages, microglia, dendritic cells and astrocytes) [2,3]. Four patterns of active lesions were described, characterized by different immunological involvement and glial and neuronal injury. Interestingly, these features were found to differ between patients but to be identical in the same individual. These findings support the important concept that different pathogenic mechanisms and targets may underlie the development of MS lesions in different patients [4,5].

During the last few decades, the concept of MS pathogenesis was profoundly influenced by research conducted in experimental autoimmune encephalomyelitis (EAE). These animal models mimic many pathological aspects and all clinical forms of MS [6,7,8]. This has led many authors to suggest that MS has a primary autoimmune etiology. Supporting this hypothesis, studies on EAE were crucial for the development of the majority of current MS treatments. These drugs have the adaptive immune system as the main target of mechanism of action and reduce the rate of relapses in most patients. However, their efficacies in preventing disability associated with progressive MS (SPMS and PPMS) have been disappointing. It is possible that the newer and most powerful approved therapies may benefit some patients with these forms of the disease but considerable concern exists regarding their toxicity and serious adverse reactions [9,10,11]. In short, current available therapies confirm previous clinical evidence suggesting that disability accumulation is largely independent of inflammatory relapse activity [12]. This scenario is interpreted as supporting the so-called two-stage hypothesis of MS immunopathogenesis. This framework suggests that the adaptive immune system drives autoimmune lesions in an early stage of the disease and clinical relapses, whereas an innate immune system dysfunction predominates in a second progressive and neurodegenerative stage independently of relapses [13].

MS is not just a white matter disease. It has been known for a long time that grey matter, which, besides myelin, is enriched in neuronal cell bodies and synaptic structures, is also affected [14,15]. Recent studies indicate the presence of inflammatory grey matter lesions of demyelination and neuronal damage during the earliest phases of the disease, which are, at least in part, independent of white matter lesions. Most importantly, the severity of grey matter atrophy and neuronal damage is the major correlate of disability progression [15]. These findings are consistent with the view that MS could be a primary neurodegenerative disorder [14]. This scenario is also in agreement with clinical data suggesting that MS, instead of being two-staged, is a one-stage disorder of progressive neurodegeneration from onset [12]. However, if MS is a chronic neurodegenerative disease from onset, myeloid cells should have a more prominent role in its pathogenesis than suggested by the two-stage hypothesis. The innate immune system could have a critical role in the regulation of autoimmunity mechanisms from the earliest states of the disease [2,16].

Immune-mediated processes are essential in the pathophysiology of MS and their qualities probably differ in relapsing and progressive phenotypes of the disease. Nevertheless, a critical issue, for which an old disagreement persists, concerns the mechanisms that initiate the inflammatory nature of the disorder [15,17]. Some authors believe that MS is caused by a primary peripheral immune dysfunction leading to autoimmune mechanisms of CNS damage (the “outside-in” model) [3,13]. An alternative hypothesis suggests that the inflammatory reaction is secondary to an abnormal development or degenerative damage of oligodendrocyte-myelin complex or/and neurons (the “inside-out” model) [14,18,19,20]. Two points should be emphasized in this context. First, there is a physiological cross-talk between the immune system and the CNS [21]. Neurons and glial cells and the peripheral immune system share the expression of many molecules, including human leukocyte antigens (HLA), complement proteins, cytokines and neurotransmitters [15,21]. Myeloid cells (macrophages and microglia) and astrocytes are involved in maintaining neuronal homeostasis, synaptic development and plasticity and myelin remodelling [2,16,22]. Regulatory T cells (Treg) are impaired in MS [3] and also possess neurotrophic proprieties and promote myelin production independently of immunomodulatory functions and overt inflammation [23]. Consequently, it would not be unexpected to find a physiological disturbance concomitantly expressed in the peripheral immune system and the CNS. Indeed, many neuropsychiatric and classic primary neurodegenerative disorders, such as Alzheimer’s disease, are associated with an abnormal systemic and brain inflammatory reactivity. Secondly, the “outside-in” and “inside-in” models are not necessarily in conflict. As outlined above, MS is clinically and pathologically a heterogeneous disease. Therefore, the pathways inducing or maintaining the abnormal immune reactivity in MS do not need to be the same in all individuals or forms/stages of the disease. These points are further discussed in this manuscript in the context of recent studies that suggest an abnormal metabolism and an involvement of peroxisome proliferator-activated receptors (PPAR) in the pathophysiology of MS.

## 2. Genes and the Environment in MS

As in many other complex diseases, the cause of MS in most patients is thought to be due to an interaction of multiple potential genetic and environmental factors. Large genome-associated studies (GWAS) identified more than 200 genetic variants associated with MS susceptibility. The HLADRB1*1501 haplotype is the most significant genetic risk factor for the MS and possibly promotes a more severe course of the disease [3,5,15]. The genes encoding the HLA molecules are only a minority of the over 250 expressed genes in the extended major histocompatibility complex (xMHC), which are mainly related to immune functions. However, disease-associations have been found in more than 100 loci outside the xMHC. A recent study identified 60 genes shared by MS and cardiovascular disease (CVD) risk factors [24]. Multiple loci in the xMHC region were overlapping between MS and triglyceride and high density lipoprotein (HDL) cholesterol while the polygenic overlap between MS and low-density lipoprotein (LDL) cholesterol and some other CVD factors was not dependent on the xMHC region. On one hand, these results are in line with the involvement of immune-mediated processes in vascular diseases; and, on the other hand, they suggest genetic influences on lipid metabolism shared by the pathogenesis of CVD and MS. Supporting this scenario, a recent large study concluded that genetically increased body mass index (BMI) is also associated with the risk of MS [25]. These results are in accordance with previous studies linking obesity in childhood or adolescence with increased risk for the disease. Multiple environmental or life style factors have been associated with the risk and severity of MS [26,27]. However, the impact of such environmental factors for the risk of MS seems greater if exposure occurs before 15 years of age; and interactions with individual and ethnic genetic backgrounds could contribute to explain the disparity of some studies regarding the influences, for example, of Epstein–Barr virus, tobacco use or salt intake [28,29,30]. While regions of higher latitude and decreased levels of sunlight exposure have higher prevalence of MS, genetic influences on the vitamin D level or action could be important [31]. Some genetic and environment factors have been found to be protective of MS [3,27]. In short, although the development of MS requires in most cases an exposure to environmental factors, even in most of these cases the disease probably occurs only in genetically susceptible individuals [32]. It should be emphasized that neither the genetic nor the environmental factors associated with the risk and clinical course of the disease need to be the same in different patients.

In a remarkable paper published in 1965, R.H.S. Thompson reviewed the large biochemical and epidemiological data implicating a disturbed phospholipid metabolism and essential polyunsaturated fatty acid (PUFA) deficiency in MS pathogenesis [33]. Notably, these metabolic alterations were interpreted to support the contribution of an abnormal brain chemical composition and vascular/anoxia mechanisms to the genesis of MS lesions. Later, Goldberg (1974) suggested a link between vitamin D and calcium deficiency and an abnormal lipid metabolism in the aetiology of the disease, which could affect the development and stability of myelin [34,35]. In the 1970s, Swank [36] reported the benefit of treating MS patients with a low fat diet for more than 20 years and epidemiological studies confirmed a correlation between high saturated animal-fat and low PUFA intake and the prevalence of MS [37,38]. More recently, a large cross-sectional study found strong associations of plant-based ω-3 supplementation (but not fish oil intake) with lower disability and relapse rate [39]. These findings are in agreement with prospective studies supporting a link of lower PUFA intake (especially α-linolenic acid) with increased risk of MS [40] and high saturated fat and low vegetable intakes with relapse risk in paediatric MS [41]. The protective effects of fatty fish intake described by some studies could be confined to individuals exposed to low ultraviolet radiation and vitamin D deficiency [42]. A very recent large cross-sectional survey in 6989 patients concluded that a high intake of fruits, vegetables and legumes and whole grains and a low intake of sugar and red meat were associated with lower levels of disability in people with MS [43].

In summary, wide clinical and epidemiological data support the view that an interplay between genetic and environmental or life-style factors affecting lipid and energetic metabolism is implicated in the development and clinical course of MS. These data suggest pathogenic links between MS and vascular diseases.

## 3. Genesis and Repair of MS Lesions

### 3.1. Demyelination Lesions

The landmark work by Shore et al. [44] in EAE concluded that “major changes in ApoE-containing lipoproteins are undoubtedly significant in the altered immune function in EAE”. This paper was followed by increased research on the role of lipids in the genesis of lesions and clinical course of EAE and MS. Newcombe et al. [45] suggested that, in MS patients, plasma low density lipoprotein (LDL) enters the CNS parenchyma as result of BBB increased permeability and is then largely oxidized and taken up by infiltrating macrophages and microglia. This mechanism is thought to contribute to the activation of these cells and phagocytosis of myelin. Oxidized phospholipids were identified in myelin, oligodendrocytes and neurons, and may be involved not only in active demyelination but also in neurodegenerative lesions [46]. However, ingestion of myelin by foam myeloid cells change their pro-inflammatory (M1) to an anti-inflammatory (M2) phenotype, which could downregulate the development of lesions and promote repair [47]. The low-density lipoprotein receptor-related protein1 (LRP1) is implicated in myelin phagocytosis and also controls BBB permeability and immune activation [48,49]. Myelin is especially enriched in sphingomyelin and other ceramide-derived compounds that are liberated during the destructive process and can be found in the CSF of MS patients [50]. However, as in other neuroinflammatory processes, astrocytes are the main origin of the increased levels of ceramide found in MS active lesions, which promotes leukocyte migration across the BBB [51]. Higher levels of palmitic acid-containing hexosylceramide (Cer16:0) in CSF were found in CIS patients who converted to MS within three years from sampling [52]. In contrast, the ceramide metabolite shingosine-1-phopsphate (S1P), among other effects, can signal endothelial cells and astrocytes to reduce leukocyte transmigration and CNS inflammatory activity. Fingolimod, a modulator of the S1P receptor, attenuates the BBB dysfunction induced by ceramide, which might contribute to its beneficial effects in MS patients [51,53]. As in plasma, S1P in the brain is mainly associated with high density lipoproteins (HDL) and may mediate some of its protective and anti-inflammatory effects [54]. Interestingly, S1P was found to be increased in the CSF of RRMS patients who were still not treated with immunomodulatory agents, supporting an involvement of ceramide metabolism from the earliest stages of the disease [55]. Myelin proteins and lipids are recognized antigenic targets of the adaptive immune system. For example, intrathecal synthesis of lipid-specific antibodies were correlated with increased relapses and a more aggressive disease [56]. However, it should be emphasized that brain phospholipids and glycolipids may also resolve inflammatory reactivity, including by suppressing activation and inducing apoptosis of autoreactive T cells [57,58].

In adult CNS, neurons and oligodendrocytes are largely dependent on cholesterol delivered by astrocytes. Apolipoprotein E (ApoE) in the brain is mainly expressed by astrocytes and necessary for the transport of cholesterol to neurons and oligodendrocytes through HDL-like particles [59]. Recently, a decrease in expression of cholesterol synthesis genes was found in chronic EAE and MS lesions and related to inflammatory infiltrates [60]. Lavrnja et al. [61] observed an altered expression of cholesterol metabolism-related genes during the development of demyelinating lesions in EAE. This work suggests that an increased cholesterol synthesis and expression of ApoE occurs in later stages of the destructive process, contributing to the regeneration of myelin and neurons. ApoE has immunosuppressive effects, inducing the differentiation of macrophages into an anti-inflammatory phenotype [62]. However, phagocytosis of myelin cholesterol and its oxygenated metabolites (oxysterols) may induce regenerative and protective mechanisms as well. Mailleux et al. [63] have shown that oxysterols are also present in myelin and that foam phagocytes generate 27-hydroxycholesterol in MS lesion. These authors observed that uptake of oxysterols by foam cells induces the expression of ApoE and other liver X receptor (LXR) genes, and stimulates anti-inflammatory mechanisms. LXR activation decreases disease severity, Th17 polarization and IL-17 secretion in EAE, though certain oxysterols may have pro-inflammatory effects in these models [64]. The Mailleux group also reported that in EAE, LDL receptor deficiency attenuates the severity of the disease in females (not in males) mice, through the induction of ApoE [65]. Interestingly, other authors have found that, in ApoE knock-out mice, ApoE deficiency increases EAE severity only in female animals [8]. These findings suggest interactions between sex steroids and cholesterol metabolism in the development of CNS lesions and severity of the disease. Sex steroids have anti-inflammatory and neuroprotective effects and the CNS is not only a target of these bloodstream hormones because it also produces sex steroids (neurosteroids), mainly by astrocytes. Luchetti et al. [66] observed that oestrogen signalling is induced in male and progesterone signalling is increased in female brain lesions of MS patients. Importantly, different alterations in sex steroid metabolism were detected even in normal-appearing white matter (NAWM). These data indicate that sex steroids could mediate gender differences associated with the genesis and repair of MS lesions.

### 3.2. Neurodegeneration and Progressive MS

R.H.S. Thompson has suggested that “…chemical differences may exist in apparently unaffected areas of brain tissue in multiple sclerosis. Such differences, possibly inborn in nature, might render the central nervous system more sensitive to other potentially damaging factors” [33]. Several studies have detected an abnormal lipid composition of NAWM that could precede myelin and neuronal inflammatory damage [18,33,35]. The Moscarello group observed that an abnormal maturation of myelin basic protein (MBP) could also contribute to the activation of autoimmune mechanisms [18]. Recent spectrometry analysis of NAWM and normal and normal-appearing grey matter (NAGM) from MS patients support the view that an abnormal brain chemical composition could precede the development of immune-mediated lesions [67]. The last study found an increased lipid peroxidation in white matter and a pattern of composition in NAWM and NAGM, suggesting a metabolic disturbance leading to a decrease of sphingolipids and increase of phospholipids content that could destabilize myelin. In this line of thought, Vidaurre et al. [68] found increased levels of ceramide C16:0 and C24:0 in the CSF of MS patients, without changes of cytokines levels. Ceramide compounds were sufficient to induce mitochondrial dysfunction, increased expression of genes involved in oxidative damage and glutamate excitotoxicity and decreased expression of neuroprotective genes. Other authors found that high levels of hexosylceramide C16:0 in the CSF correlated with disability scores only in progressive patients [52]. Increasingly data support major roles of mitochondrial dysfunction, reactive oxygen and nitrogen species, glutamate excitotoxicity and ion channel dysfunction (mainly of calcium homeostasis) in the neurodegenerative process and progression of the disease [69,70,71]. Accordingly, *N*-acetyl aspartate, a metabolite only produced by neuronal mitochondria and required for myelin synthesis is decreased in NAWM and NAGM and correlated with disability [72,73]. Reduced oxygen consumption (hypoxia) and age-dependent brain iron accumulation may amplify oxidative damage and the neurodegenerative process. Local cyclooxygenase-dependent lipid oxidation metabolites were also suggested to contribute to the mechanisms of disease leading to disability progression [74]. The transcriptional factor (erythroid-derived2)-like 2 (Nrf2), a target of dimethyl fumarate therapy in MS, is activated by excessive free radical production, induces the expression of many antioxidant defenses and is upregulated in inflammatory lesions of the disease [75]. A diminished expression of Nrf2 was correlated with reduced levels of glutathione in EAE [76] and a marked reduction of glutathione was recently observed in brains of progressive MS (SPMS and PPMS) in comparison to RRMS patients [77]. It should be noted that neuron–astrocyte interactions may have major roles in the regulation Nrf2 signalling in the CNS [78]. In a chronic pro-inflammatory milieu, diminished synthesis of cholesterol by astrocytes critically compromise myelin remodelling and maintenance of neuronal structural integrity [59,60]. Several studies have shown a correlation between neuronal damage and brain atrophy and decreased synthesis of 24S-hydroxycholesterol (24OHC), which mainly occurs in neurons [79]. An additional element of complexity concerns the complement proteins, which in the adult human brain are mainly synthesized in neurons and have physiological roles in synaptic elimination and remodelling. As in the immune system, these proteins may opsonize cellular components for clearance by activated macrophages and microglia and could drive synaptic loss from the earliest stages of MS [80]. Recent work observed a widespread and pronounced synaptic loss in the cerebral cortex of MS patients independent of cortical demyelination and axonal loss [81]. Neuronal dysfunction could be a source of local complement production independent of systemic circulation and genesis of acute demyelination lesions and related with the progression of the disease [82]. Again, astrocytes constitute important players in this scenario. Cytokines and complement from activated microglia change astrocytes to a “reactive” and toxic phenotype, inducing synaptic loss and death of neurons and oligodendrocytes [83]. An increased synthesis and release of lactosylceramide by astrocytes was found in EAE and MS lesions, promoting the recruitment and activation of monocytes and microglia and neurodegeneration [84]. S1P receptors in astrocytes control the development of acute lesions and nuclear factor-kappa B (NF-κB) activity associated with CNS inflammation in chronic progressive EAE and MS [85]. Kynurenine acid (KA) is also produced in the brain mainly by astrocytes, has anti-inflammatory and antioxidant effects and protects neurons against glutamate toxicity. Abnormalities in the kynurenine pathway of tryptophan metabolism possibly have important roles in the neurodegenerative mechanisms of MS (71) (see below). In addition, cortical and meningeal infiltrates of lymphocytes are frequently present in progressive disease and may contribute to grey matter and neurodegenerative pathology as well [15,70].

In summary, it is generally accepted that white matter focal demyelination and axonal lesions are mainly driven by immune cell infiltration from the periphery, whereas a compartmentalized diffuse microglial and astrocyte activation mainly drives grey matter and synaptic loss pathology and progressive MS [3,15]. Nevertheless, myeloid cells and astrocytes are critical players in all pathogenic processes of MS [2]. Furthermore, alterations in lipid, oxidative and other metabolic pathways are present in the CNS from the earliest stages of the disease and are involved in mechanisms modulating immune activation and the development and repair of demyelinating and neurodegenerative lesions.

## 4. Systemic Metabolism in MS

### 4.1. Plasma Lipids

Giubilei et al. [86] were the first to report a correlation between the number of new brain lesions evaluated by magnetic resonance imaging (MRI) and the mean plasma level of total and LDL cholesterol in patients with the first clinical episode suggestive of MS (CIS). Recent prospective studies have variably found associations between total cholesterol (TC), LDL, non-HDL, TC/HDL, triglycerides, apolipoprotein B and the risk of new lesions accumulation and disability progression in patients with RRMS and/or CIS. Some studies also reported correlations between higher plasma ApoE levels and severity of EAE, higher disability in RRMS, and deep grey matter atrophy in CIS patients. In contrast, higher levels of HDL and of its major apolipoprotein, ApoA1, were associated with lower blood–brain barrier permeability and protection to the development inflammatory lesions [87]. RRMS subjects have smaller LDL in comparison to healthy controls and in some cases increased levels of small HDL with impaired anti-inflammatory activity [88]. Interestingly, this study observed some differences between male and female patients, suggesting gender differences in lipid metabolism associated with MS [65]. Supporting this hypothesis, recent research from our group suggests that sex steroids modify the serum lipid profile associated with disability in these patients (unpublished results). Plasma oxysterols levels are increased in RRMS and mainly associated with small dense LDL, supporting a link with mechanisms promoting atherogenesis [89]. These data disclose many evident similarities between MS and the mechanisms involved in atherosclerotic plaque development and progression, as mentioned by Ludewig and Laman [90]. In their paper, these authors concluded that…”Systematic comparison of these two diseases involving foam cells in chronic lesions may prove fruitful”. Besides hypercholesterolemia, the coexistence in the MS patient of other related vascular pathology (such as diabetes and hypertension) may indeed be associated with more rapid disability progression [91] and increased risk of relapses [92]. Taken together, the results from these studies and those summarized in the above sections strongly suggest that CVD and MS may share certain pathophysiological mechanisms [93]. Statins have well-known immunosuppressive proprieties and decrease inflammatory activity and clinical signs in EAE. In 2003, our group published a pilot study suggesting potential benefits of lovastatin monotherapy in RRMS [94] and similar results were obtained by Vollmer et al. [95] using simvastatin. More recently, simvastatin was shown to reduce brain atrophy and to improve cognitive and physical quality of life measures in SPMS patients [96,97]. Interferon beta therapy changes the associations between serum lipoprotein levels and the clinical activity and neurodegenerative process of the disease [98,99]. Current approved drugs for MS may induce specific alterations in systemic lipid metabolism. In one study, interferon beta was shown to increase subspecies of ceramides and natalizumab to increase S1P and sphinganine-1 phosphate, whereas fingolimod did not affect the levels of these lipids in plasma [100].

Systemic metabolic alterations in MS must have characteristics unique to the disease and to its different pathological and clinical phenotypes. Metabolomic investigations have found distinctive serum phospholipid and sphingolipid patterns in MS patients in comparison to healthy controls subjects or patients with other neurological disorders [101,102,103]. In one study, an increase of phospholipids and a decrease of sphingolipids were observed and certain phospholipids, glutamic acid, tryptophan and arachidonic acid metabolites levels were correlated with a more severe disease [102]. In addition, Quintana et al. [104] reported different patterns of serum antibodies to lipids and other CNS antigens associated with RR, SP and PP forms of MS and Bakshi et al. [105] found that serum lipid antibodies associated with atrophy differed from those associated with brain focal lesions. Some authors believe that this profile of alterations in the serum of MS patients is mainly due to the chronic activation of the immune system [102]. However, an underlying abnormal metabolism could also affect immune reactivity. Obviously, these two mechanisms are not mutually exclusive and their role could differ, depending on the individual patient and activity/type of the disease. We have found that in patients displaying similar clinical activity and disability scores, lower serum ApoE levels were associated with the increased risk for development of neutralising antibodies to interferon beta [106]. These results are consistent with anti-inflammatory effects of ApoE [62,63,107] and suggest that individual differences in lipoprotein metabolism could influence the reactivity of the immune system in these patients [106].

### 4.2. Metabolism and Immune Dysfunction

It is presently indisputable that specific metabolic processes are needed to support the different functions of immune cells. Distinct metabolic programs are required for differentiation and function of effector T cells (Th1, Th2, Th17) and inducible regulatory T cells (Treg) [108]. Activation and proliferation of CD4^+^ and CD8^+^ T effector cells depend on glycolysis, whereas T memory cells depend on fatty acid oxidation for ATP production [108,109]. A reduction in proliferation and suppressive functions of Treg cells is one of the main characteristics of the immune dysfunction in RRMS patients [3,110]. Although Treg cells also rely on lipid oxidation, an engagement on glycolysis may be necessary to generate the suppressive functions of these cells [111]. Interestingly, an impairment of glycolysis and mitochondrial respiration was recently observed during T cell activation in RRMS patients, which was reversed by interferon beta treatment [112]. One of the main signalling pathways that trigger glycolysis during inflammatory immune activations involves the transcriptional factor hypoxia-inducible factor 1α (HIF 1α), which is suppressed by dimethyl fumarate [113,114]. Different types of fatty acids have distinctive effects on immunity. Medium- and long- chain saturated fatty acids (LCFA) promote the differentiation of CD4^+^ T cells towards Th1 and Th17 cells and pro-inflammatory M1 macrophages, while suppressing the differentiation and function of Treg cells [114]. ω-6 PUFA may induce inflammatory reactivity, whereas ω-3 PUFA suppress innate and adaptive immune reactivity, in line with protective effects in MS [115]. In contrast, short chain saturated fatty acids (SCFA) promote Treg functions and suppress Th17 inflammatory activity (see below). Proliferation of immune cells require the generation of nucleotides, cholesterol and specific fatty acids, lipids and proteins. The glycolytic derived pentose phosphate pathway allows the production of nucleotides and the reduced form of nicotinamide adenine dinucleotide phosphate (NADPH) which is used for fatty acid synthesis and to generate glutathione and other antioxidants. Fatty acid synthesis is especially needed for differentiation and inflammatory functions of M1 macrophage, dendritic cells and effector T cells [114]. Certain sphingolipids, apolipoproteins and amino acids (such as glutamate and tryptophan) also have specific roles in the signalling or transduction mechanisms of immune cells [64,114,116] . In particular, tryptophan derived metabolites of the l-kynurenine pathway, among many other compounds in circulation, regulate inflammation through the aryl hydrocarbon receptor (AHR) in immune cells and astrocytes [117]. RRMS patients have lower serum levels of AHR agonists in comparison to healthy controls. However, increased AHR agonists levels were detected during acute CNS inflammation, probably reflecting an attempt to restrict immune activation [118]. In addition, an abnormal l-kynurenine metabolism was linked to the development of progressive forms of MS [71,119].

Steroid hormones, insulin, leptin and adiponectin all have distinctive modulatory roles on immune cells functions [120]. An impairment of insulin signalling following CD4^+^ and CD8^+^ effector T cells activation attenuates the symptoms in EAE [121] and alterations in peripheral insulin sensibility have been described in RRMS patients [88,92,122]. In MS patients who developed metabolic syndrome, metformin treatment decreases serum leptin and increases adiponectin levels. A decreased secretion of pro-inflammatory cytokines by peripheral blood mononuclear cells (PBMC) and increased Treg number and function was also observed after metformin treatment [122]. In EAE, a diet mimicking fasting promotes remyelination and a lower pro-inflammatory state and increases in plasma levels of corticosterone and adiponectin [123]. In contrast to leptin, adiponectin has anti-inflammatory protective roles in MS possibly mediated by ceramide metabolites, such as S1P [116,120]. Leptin-deficient genetically obese mice (ob/ob) present an altered systemic and brain development, including in the amount and fatty acid composition of myelin [124]. Interestingly, although these mice are congenitally resistant to EAE induction, exogenous leptin replacement render these animals susceptible to the disease [120]. These findings support the view that alterations induced in systemic metabolism may have critical roles in promoting the development of MS, even in individuals not genetically susceptible to the disease.

The hypothesis that an “enteropathy” [33] or a defect in the metabolism of “some component of westernized diet” [17] could contribute to the development of MS were discussed many years ago. During the last decade, the evidence for important physiological roles of gut microbiota (GM) in regulating the immune functions has revisited and illuminated those old, intriguing hypotheses. These regulatory effects are mediated through microbiota-dependent alterations in hormonal levels and metabolism of carbohydrates, lipids and amino acids [125,126,127,128]. Sex differences in the GM result in altered serum levels of testosterone and of glycerophospholipids and sphingolipids, which could contribute to the increased susceptibility of females to MS [129] . On one hand, GM is required for the induction of autoimmune T and B cells demyelination in EAE [130]. On the other hand, GM produces SCFA (acetate, butyrate, and propionate) from dietary polysaccharides, inducing the generation of Treg cells and protection against the disease [131]. Butyrate was also shown to downregulate innate response receptors in human monocytes [132]. Dietary LCFA enhances differentiation and proliferation of effector T cells and exacerbates the symptoms of EAE, whereas administration of SCFA reduces Th1 and increase regulatory T cells and is protective [133,134]. Alterations in GM composition promoting a pro-inflammatory milieu have been observed in MS patients regardless of immunomodulatory treatments and associated with clinical relapses, suggesting a decrease in fatty acid metabolism and engagements in defence pathways linked to oestrogen signalling and production of bile acid metabolites and glutathione [135,136]. Notably, transplanted GM from MS patients promotes the induction or exacerbates EAE symptoms in association with reduced proportions of interleulin-10 (IL-10) secreting Treg cells [137,138]. High frequency of intestinal Th17 cells in MS patients was correlated with decreased abundance of Prevotella strains and increased activity of the disease [139]. Treatment with interferon beta or glatiramer acetate raises the quantity of Prevotella and other current approved drugs for the disease and vitamin D supplementation could change microbial intestinal composition as well [125,126,127,128]. GM may regulate BBB permeability [140], brain myelination [141], microglia and astrocyte activities [117,142]. In humans, dietary ω-3 fatty acids change microbiota composition and induce the production of anti-inflammatory compounds like butyrate [143]. Pilot trials in MS suggest that a ketogenic diet normalizes the mass and diversity of the colonic microbiome [144] and that the modulation of dysbiosis by a high-vegetable/low protein diet is associated with an increase of Treg differentiation and IL-10 production and improvement in clinical courses [145]. Several strategies to manipulate GM are presently being considered for therapeutic proposes in MS [127]. Recent studies suggest that alterations in GM could mediate the possible influences of salt intake on the risk and progression of the disease. A high salt diet affects the composition of mice GM, inducing Th17 cells, the development of EAE, hypertension [146] and cognitive dysfunction [147].

In short, the studies reviewed here and in the sections above indicate that the abnormal immune reactivity in MS can be modulated by genetic and environmental factors shaping the metabolic and hormonal milieu of the organism. These metabolic processes may either result in loss of immune tolerance to self and drive the induction the disease or be protective of its development and progression. In this context, Peroxisome proliferator-activated receptors (PPAR) may comprise important players in MS pathogenesis, as discussed below.

## 5. Peroxisome Proliferator-Activated Receptors (PPAR) in MS

PPAR are transcriptional factors involved in the regulation of lipid and glucose metabolism, cell differentiation and proliferation. The PPAR subfamily of nuclear receptors comprise the members PPARα (NR1C1), PPARβ/δ (NR1C2) and PPARγ (NRC1C3), which, after ligand activation, regulate gene transcription by dimerizing with the retinoid X receptor and acting in specific DNA sequences. In addition, PPAR can repress gene expression in a DNA-binding independent way by interfering with other transcription factors. PPAR can be activated by synthetic agonists and specific endogenous ligands, mainly PUFA and eicosanoid metabolites [148,149,150]. All PPAR subtypes are variably expressed in immune cells and have important roles in the control of innate and adaptive immune functions [151,152,153]. Several studies have shown protective effects of PPAR agonists in EAE [154]. Current data suggest that each PPAR isoform is able to control the development of T cell- mediated autoimmunity in EAE by distinctive mechanisms. PPARα promotes Th2 cells differentiation and cytokines production [155], whereas PPARγ strongly restricts Th17 differentiation [156] and PPARβ/δ expression inhibits the production of interferon-γ (INF-γ)and IL-12 [157]. PPARγ could be a major driver of Treg cell accumulation and functioning [158]. Accordingly, administration of eicosapentaenoic acid in the diet ameliorates the clinical symptoms of EAE. Importantly, although this treatment slightly increased only PPARγ in the periphery, all PPAR isotypes were induced in CNS-infiltrating CD4 T cells [159]. In the CNS, PPAR activation inhibits the NF-κB and Janus kinase-signal transducer and activator of transcription (JAK-STAT) pathways of a complex network involving adaptive immune cells, myeloid cells and astrocytes [151,160]. All subtypes of PPAR may regulate astrocyte Toll-like receptors stimulation in inflammatory responses [161]. PPAR activation is in general inhibitory of innate immune cells activation and foam cell formation, promoting macrophage polarization to an anti-inflammatory phenotype [149,152]. PPARγ activation in the CNS during EAE reduces the production of inflammatory and neurotoxic mediators by macrophages and astrocytes and re-stimulation of infiltrating autoreactive T cells, supporting a major role in the control of disease progression. These findings suggested that endogenous PPARγ ligands, namely induced by IL-4, could inhibit the formation of foam cells and CNS autoimmunity [162]. In EAE animals and active MS lesions, specific activation PPARβ/δ was also able to promote an anti-inflammatory phenotype in macrophages after uptake of myelin lipids and foam cell formation, decreasing immune cell infiltration in the CNS [163].

MS patients present an impairment of oligodendrocyte precursor cells (OPC) differentiation compromising remyelination, which is increasingly deficient with the progression of the disease. Selective PPARβ/δ agonists stimulate oligodendrocyte differentiation [164]. Gemfibrozil, an activator of PPARα often prescribed to lower triglycerides levels, was shown to increase the expression of myelin genes (including myelin basic protein, MBP and myelin oligodendrocyte glycoprotein, MOG) in human oligodendrocytes via PPARβ/δ (not via PPARα or PPARγ) [165]. PPARγ agonists induce astrocyte and prominently OPC differentiation genes of neural stem cells,, though not MBP and MOG [166]. PPARγ activators further promote OPC maturation toward myelin-forming oligodendrocytes, increasing MBP expression and membranes enriched in cholesterol and plasmalogens [167,168,169]. Promotion of myelinogenesis by PPARγ signalling involves an increase of antioxidant defenses and mitochondrial respiratory activity [168,169]. Importantly, docosahexaenoic acid and others’ endogenous and synthetic PPARγ activators may protect against the impairment of oligodendrocyte maturation and myelination associated with inflammatory conditions [170,171]. The mechanisms promoting remyelination in MS by PPAR could involve downregulation of NF-κB/β-catenin and activation of PI3K/Akt pathways [172].

PPAR are expressed in all cells of adult mouse and human brains, PPARβ/δ being the most abundant [173,174]. Curiously, all PPAR isotypes are mainly expressed in neurons. In normal conditions, it was observed that PPARβ/δ is only expressed in neurons, whereas PPARα colocalizes in neurons, astrocytes and microglia. PPARγ colocalizes in neurons and astrocytes, but its expression could be induced in microglia after LPS treatment [174]. These recent observations highlight the importance of interactions in the PPAR triad for the CNS physiology and pathology accordingly with distinctive roles of these three isomorphs in immune function and myelin formation. In the CNS, PPARβ/δ probably has a major integrative role and was suggested as the main target to be considered in therapeutic strategies for neurodegenerative diseases [175]. PPARβ/δ agonist GW0742 promotes neuronal maturation in parallel with increased expression of brain-derived neurotrophic factor (BDNF) and activation of cholesterol synthesis [176]. Nevertheless, selective agonists of other PPAR subtypes also induce neuroprotective effects in vitro and in animal models of neurodegenerative disorders [177]. Recent research supports an essential role of these nuclear receptors in brain development and suggest that individual variability in PPAR signalling may be associated with susceptibility to various neurological insults and diseases [178]. In parallel with anti-inflammatory and remyelination effects, many studies have demonstrated protective effects of PPAR agonists against central players in the neurodegenerative process in MS and other disorders, including mitochondrial dysfunction, calcium dysregulation, glutamate toxicity and oxidative injury [175,177]. For example, PPARγ agonists have protective effects on glutamate excitotoxicity by increasing the expression of the major transporter (GLT-1) of this neurotransmitter in astrocytes [179]. As mentioned above, Nrf2 signalling and glutathione availability are compromised very early in the disease. Deficiency in glutathione induces macrophage CD36 expression at a translational level, independently of PPARγ activation, resulting in enhancement uptake of oxidized LDL and foam formation [180]. PPARγ and Nrf2 are linked by a positive feedback loop that sustains the expression of both transcriptional factors and antioxidant defences [181]. PPARγ is a critical mediator on the effects of galangin (a polyphenolic abundant in honey and certain vegetables) in decreasing NF-κB activation and increasing Nrf2 signalling in LPS-stimulated microglia [182]. PPARγ agonists have protective effects on mouse cochlea against gentamicin toxicity through anti-apoptotic effects and upregulation of glutathione and other antioxidant defences [183]. Interestingly, this work revealed some protective effects of fenofibric acid (a PPARα-specific agonist) by affecting different antioxidant mechanisms. Decreased neuronal concentrations of glutathione stimulates the generation of 12/15-lipoxygenase metabolites and the inhibition of this pathway by baicalein (a type of flavonoid) induces the expression of PPARβ/δ in microglia and attenuates inflammation in EAE [184]. Recent studies suggest protective effects of α-lipoic acid (an endogenous antioxidant) therapy on the neurodegenerative process of EAE and MS [185], which could be mediated by PPARγ activity [186]. In brief, a huge body of studies in vitro and in animal models indicate that all PPAR subtypes could be involved in the control of immune activity and mechanisms promoting myelination and neuronal protection in MS by affecting different but complementary cellular and molecular pathways.

The Heneka group was the first to investigate PPAR signalling in MS patients. In one study, it was shown that peripheral blood mononuclear cells (PBMC) from these patients expressed decreased PPARγ levels correlated with disease activity and preceding the development of clinical relapses. Moreover, pre-treatment with the PPARγ agonist pioglitazone was able to increase PPARγ DNA-binding and decrease NF-κB DNA-binding activity in patients in a stable stage of the disease. These results indicate a suppression of PPARγ by inflammatory stimuli and that PPARγ agonists are protective through increased expression of their own receptors [187]. Preliminary results suggest an influence of PPARγ gene polymorphism in modulating the onset of MS [188], and beneficial effects of pioglitazone treatment in reducing the development of brain lesions and grey matter atrophy and clinical progression of the disease [189,190,191]. A pronounced elevation of PPARγ levels was observed in the CSF of MS patients free of therapy, which were correlated with intrathecal inflammatory parameters and clinical disability [192]. In accordance with the results reviewed above, these findings could reflect an attempt of PPARγ-mediated processes of brain cells in restricting immune activity. A crucial and very early event in MS pathogenesis concerns the abnormal leucocyte transmigration to the CNS across the BBB, a process promoted by inflammatory activation of endothelium and involving integrin β1-mediated mechanisms. PPARγ was shown to inhibit leucocyte migration across activated brain endothelial cells [193]. Interestingly, ω-3 PUFA increases PPARγ expression and decreases the expression of integrin β1 in glomerular mesangial cells treated with LPS [194]. PPARγ is a well-known inductor of CD36 receptor expression. This scavenger receptor requires interactions with integrins for many of its functions [195], and may regulate BBB permeability [196]. Natalizumab (NTZ) treatment blocks α4/β1 integrin resulting in peripheral sequestration of activated T cells and increased production of proinflammatory cytokines. In female patients, we have found an increase of PPARβ/δ mRNA and a decrease of PPARγ and CD36 mRNA expression in PBMC at three months of NTZ therapy. In contrast, PPARα was unchanged [197]. Interestingly, inflammatory activity of monocyte derived human macrophages is associated with lower PPARγ/CD36 and higher PPARβ/δ expression levels [198]. In addition, PPARβ/δ agonists may stimulate pathways enhancing immune reactivity under hypoxic stress, suggesting context-dependent functions [199]. These selective responses support the concept that PPAR subtypes may have reciprocal or complementary roles in immune regulation. Fingolimod (FTY720) is an S1P mimetic that down-modulates S1P signalling, retaining central memory T cells and B lymphocytes in lymph nodes and preventing autoreactive cell infiltration in the CNS [200]. In contrast to NTZ, fingolimod treatment decreases the number of reactive lymphocytes in circulation. Preliminary results from our group have found that, at six months of therapy, fingolimod augmented PPARγ and CD36 mRNA expression in total blood leucocytes from female patients, whereas PPARβ/δ and PPARα were unaffected. Importantly, this selective alteration in PPARγ/CD36 mRNA expression was associated with a significant increase of plasma HDL levels and decrease of total cholesterol/HDL ratio [201]. As mentioned above, S1P is mainly associated with HDL particles and is involved in the control of BBB dysfunction and brain lesions in MS. Therefore, the diminished systemic immune reactivity induced by fingolimod could be related to protective effects of HDL. In fact, the antioxidant and anti-inflammatory proprieties of HDL are thought to be regulated, at least in part, by upregulation of PPARγ/CD36 pathway [198,202] As already mentioned, MS patients display an adverse lipoprotein metabolism that may be associated with a dysfunctional pro-inflammatory HDL particle affecting PPARγ/CD36/Nrf2 signalling [203]. Further work by our group is now on course to evaluate whether upregulation of PPARγ/CD36 pathway is associated with the clinical benefits of fingolimod treatment in the disease. The fact that PPARα expression was unchanged in female patients treated with NTZ and fingolimod is of considerable interest. The Steinman group has shown that PPARα is expressed at higher levels in males in comparison to female naïve T cells through androgen-mediated effects. Higher PPARα expression in males was associated with protection to developing EAE [155]. In contrast, 17β-estradiol increases PPARγ expression, supporting evidence for an increased efficacy of pioglitazone in females [204]. However, the neuroprotective effects of the phytoestrogen daidzein were shown to be due to an increase of PPARγ activity not mediated by receptor binding and not additive with rosiglitazone [205]. PPARγ signalling has more wide and profound anti-inflammatory effects in females, selectively reducing only Th17-cell differentiation in males [206]. These data indicate gender differences associated with the roles of PPAR in the control of immune functions and neurological homeostasis [207], possibly contributing to the modulatory effects of sex steroids in susceptibility, genesis/repair of CNS lesions, and clinical courses of MS [66,208,209].

**Figure 1:**
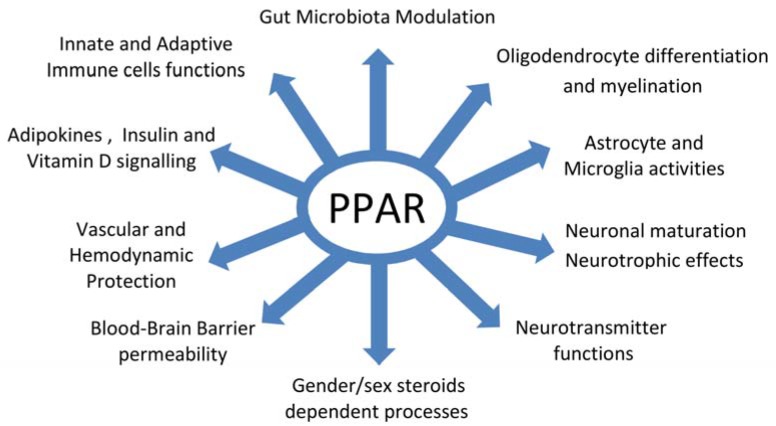
Pathophysiological pathways in multiple sclerosis potentially modulated by Peroxisome Proliferator-Activated Receptors (PPAR)-mediated metabolic processes.

PPAR-mediated processes could be implicated in other pathogenic pathways suggested for the disease as mentioned above. Vitamin D and PPARγ may interact in the mechanisms leading to differentiation of immune cells restricting pro-inflammatory activity [210]. In MS patients also developing metabolic syndrome, pioglitazone treatment decreases leptin and increases adiponectin serum levels in association with reduced secretion of pro-inflammatory cytokines by PBMC [122]. As quoted above, oxysterols’ uptake by macrophages and LXR activation is probably an important driver in repair mechanisms of MS lesions. PPAR and LXR are well known to cooperate in suppressing innate and adaptive reactivity and cell foam formation [149,152,153]. However, LXR activation may affect PPARγ-dependent expression of adiponectin, promoting inflammation and insulin resistance [211]. Therefore, LXR activation is probably not a good therapeutic approach for MS. PPAR activation may induce LXR transcription and both PPARγ and LXR agonists upregulate ApoE expression. Interestingly, PPARγ-mediated neuroprotective effects of daidzein were shown to be critically dependent of ApoE induction [212]. Finally, the increasing evidence for an involvement of gut dysbiosis in MS further supports a potential role of PPAR in its pathogenesis. The anti-inflammatory effects of microbiota derived SCFA (butyrate and propionate) or α-linolenic acid are mediated through PPARγ activation [213,214]. Microbiota-activated PPARγ signalling prevents gut dysbiotic expansion [215], and a high fat diet promotes intestinal dysbiosis, at least in part by interfering with PPARγ activation [216]. PPARα deficiency also results in microbiota-dependent increase in intestinal inflammation [217]. Intestinal dysbiosis may affect the generation of tryptophan-derived ligands of AHR in immune cells and astrocytes [118]. Butyrate and propionate are able to control AHR gene expression and activity [213]. Therefore, PPAR- dependent pathways may be involved in the regulation of systemic and brain inflammatory reactivity by AHR ligands. The main pathophysiological pathways in MS potentially modulated by PPAR-mediated mechanisms are summarized in Figure 1.

In 2011, Angelique Corthals advocated a major role of lipid dysregulation in the pathogenesis of MS, possibly involving PPAR signalling [218]. The present review emphasized that the last decade of research has revisited this topic with new insights, with the very old evidence for the importance of metabolic mechanisms in MS. Nevertheless, these advances do not allow for concluding that…”multiple sclerosis is not a disease of the immune system” [218]. The development and progression of MS only occurs in a framework of immune dysfunction. If PPAR are involved in the mechanisms of disease, it will be mainly due to the fact, that, among other roles, they are important modulators of the innate and adaptive immune functions. Notably, metabolic pathways modulated by PPAR could integrate the concomitant development of many systemic and neurological pathological processes in the disease. In addition, this scenario is coherent with much clinical and epidemiological data. However, most evidence to date is based on studies in vitro and in animal models of MS and much more research needs to be performed in human patients. As MS is pathological and clinically heterogeneous, and is associated with multiple potential genetic and environmental risk factors, PPAR-dependent processes may not necessarily be implicated in every individual and type/stage of the disease. As in other areas of human health, much work in the field is presently aimed to search for useful biomarkers for personalized preventive and treatment strategies of the disease, the so-called “precision medicine” [3,5,128]. In this context, PPAR deserve to be explored as potential targets to monitor the disease and to discover improved and individualized therapeutic measures. In a real-world setting, clinical evidence indicates that even the most powerful current strategies to modulate or suppress immune functions are of doubtful benefit or not tolerated in some patients, and may carry the risk of serious adverse events. Furthermore, very probably, they will never be sufficient to delay the evolution of progressive forms and the neurodegenerative process of the disease. In the future, much effort is still needed to trace the metabolic roads that may light and nourish “the fire” of immune activation in MS. Perhaps we should recognize that …”we have mistaken the smoke for the fire” [17].

## 6. Conclusions

During the last few decades, much progress has been made in clarifying the role of metabolic mechanisms in the pathogenesis of MS. In this context, PPAR-mediated processes have emerged as potential players able to integrate many systemic and CNS events occurring in the disease. These nuclear receptors could be implicated in pathophysiological pathways involving lipid, glucose, amino acid and antioxidant metabolism and the regulatory roles of insulin, adipokines, steroid hormones and gut microbiota. Importantly, metabolic roads modulated by PPAR seem to provide strategies to control the immune dysfunction and the genesis of CNS lesions of MS, and to promote neuroprotection and myelin formation. PPAR seem to constitute research targets that may lead to further therapeutic and preventive measures for this disorder.
